# Monitoring Alveolar Ridge Remodelling Post-Extraction Using Sequential Intraoral Scanning over a Period of Four Months

**DOI:** 10.3390/ijerph17186638

**Published:** 2020-09-11

**Authors:** Khaled E. Ahmed

**Affiliations:** Griffith Health Centre (G40), Office 7.59, School of Dentistry and Oral Health, Griffith University, Southport, QLD 4215, Australia; khaled.ahmed@griffith.edu.au; Tel.: +61-7-5678-0596

**Keywords:** prosthodontics, CAD/CAM, scanning, remodelling, digital, alveolar

## Abstract

The potential applications of computer-aided design/computer-aided manufacturing (CAD/CAM) and intraoral scanning exceed the delivery of standard prosthodontic interventions. The aim of this study was to clinically present a developed assessment technique, that relies on the use of sequential intraoral scanning, three-dimensional superimposition, and 2D and 3D deviation analyses based on a standardised protocol, as an auxiliary tool in monitoring dimensional changes of residual ridge post-extraction with a follow-up period of four months.

## 1. Introduction

Residual alveolar ridge remodelling post tooth extraction is a progressive process that involves a series of biological and physiological events resulting in both vertical and horizontal alveolar bone resorption, especially in the absence of ridge preservation interventions. It is dependent on a host of local and systemic factors [1]. Studies have previously used in-vivo cone beam computed tomographic (CBCT) scanning or extraoral surface scanning of patient casts to monitor and quantify bone remodelling up to four months post-extraction [2,3]. Indeed, a recent study involving ten patients demonstrated the use of CBCT to measure volumetric changes post vertical ridge augmentation in the form of vertical bone gain, planned bone volume, lacking bone volume, and regenerated bone volume, using computer-aided design/computer-aided manufacturing customised titanium meshes [4]. However, CBCT scans carry the disadvantage of radiation exposure, whereas dental stone casts undergo linear expansion over time that can significantly affect their accuracy [5]. In contrast, intraoral scanning offers a distinct advantage over conventional impressions in their reported potentials to reduce both chairside time [6] and patient discomfort [7,8]. Moreover, certain intraoral scanning systems have been reported to be significantly more accurate than CBCT [9]. Nonetheless, the accuracy of full-arch intraoral scanning remains limited when compared to bench scanning and is significantly dependent on the scanning system [10]. Although scan superimposition and deviation analyses have been successfully used in monitoring hard tissue changes over time [11] yet its application in monitoring soft tissue changes remains limited. The aim of this report was to present a developed assessment technique based on sequential intraoral scanning, three-dimensional superimposition, and deviation analyses based on a standardised protocol, as an auxiliary tool in monitoring dimensional changes in the residual ridge post-extraction over time.

## 2. Materials and Methods

**Figure 1:**
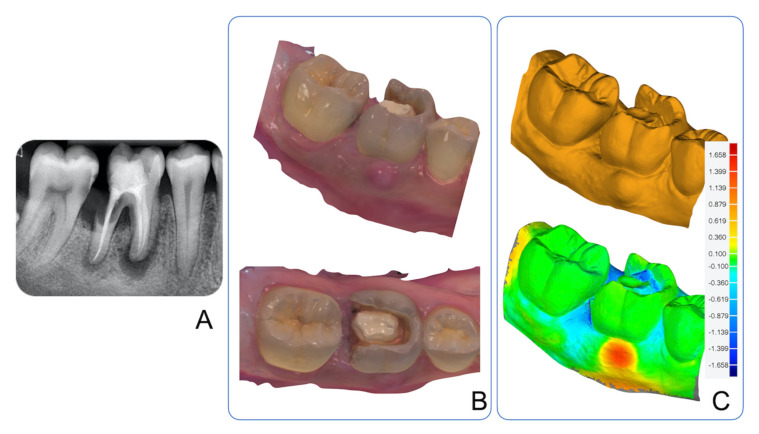
(**A**) Periapical radiograph of lower right posterior teeth demonstrating multiple periapical and radicular radiolucencies associated with endodontically treated tooth 46 and a mesial coronal radiolucency on tooth 47. (**B**) Intraoral scan of lower right posterior teeth demonstrating a dental abscess and failing restoration. (**C**) 3D-deviation analysis comparing intraoral scans pre and post abscess development.

A 37-year-old male, with no significant medical history, presented with the chief complaint of a dental abscess with a buccal discharging sinus at the site of the mandibular first molar, tooth 46 (Figure 1). The sinus had persisted for one month with mild discomfort. The tooth was endodontically treated three years ago and temporarily restored using resin-modified glass-ionomer cement (RMGIC), which the patient did not seek to definitively restore. The patient had quit smoking one year earlier and had smoked for ten years prior. Upon examination, the distal wall of the RMGIC was missing with clear marginal ditching present. Tooth 46 was slightly tender to percussion with a 5 mm probing pocket depth on the distal surface and bleeding on probing. No other clinically detectable carious lesions or periodontal pockets > 3 mm were present. Radiographically, periapical radiolucencies were noted at the mesial and distal roots and at the cervical third of the distal root extending to the furcation area of tooth 46. An additional radiolucency was present on the mesial surface of tooth 47, graded as an initial RA2/3 lesion according to the International Caries Classification and Management System (ICCMS) radiographic scoring. Given the extensive nature of caries and periapical pathology, tooth 46 was deemed to have a poor prognosis and unreasonable to restore. The patient agreed to get tooth 46 extracted and did not wish to replace it. As part of a comprehensive treatment plan, non-operative treatment of tooth 47 carious lesion and management of risk factors were completed.

### Intraoral Scanning and Quantitative Assessment of Residual Alveolar Ridge Remodelling

Prior to the extraction of tooth 46, an intraoral scan of the patient’s dentition was completed and compared to a previous scan performed two months earlier (Figure 1). Post-extraction, sequential scans were performed at days 0, 2, 3, 7, 10, 14, 21, 28, 35, 42, 56, 70, 84, and 112.

Scans were acquired using the TRIOS^®^3 colour intraoral scanner (3Shape Copenhagen, Denmark). The scanning was performed by appropriately controlling moisture using high-volume suction and cotton rolls, with cheek retractors in place, and following the manufacturer’s recommendations. The scanning protocol involved initial scanning of the occlusal surface as a reference, followed by the lingual and buccal surfaces of teeth. All scans were performed by the same experienced operator. A surface-matching software (Geomagic Control, 3D Systems, Oregon, OR, USA) was used for superimposition, followed by 3D and 2D deviation analyses of sequential intraoral scans. To minimise the iterative closest point algorithm error, several measures were deployed, including standard trimming of the scans to only include the extraction site and two adjacent teeth and removing all mobile soft tissues; using cusps of teeth 45 and 47 as reference areas; using an initial registration sample of 300 points followed by an additional iteration of 1500 points; acquiring sequential scans at frequent intervals; and performing the registration against one of the three reference scans at days 0, 7, or 28 post-extraction to minimise significant changes to hard and soft tissues. As a result, the best-fit registration involved a maximum model length of 32 mm with a root mean square error of 0.025 mm and an average error of 0.018 mm. For the step height 3D deviation analysis, the maximum critical tolerance was ±1.6 mm and the minimum critical tolerance was ±0.1 mm. For the 2D deviation analysis, three sagittal planes were made across the site of tooth 46 covering an area of 10 mm.

## 3. Results

**Figure 2:**
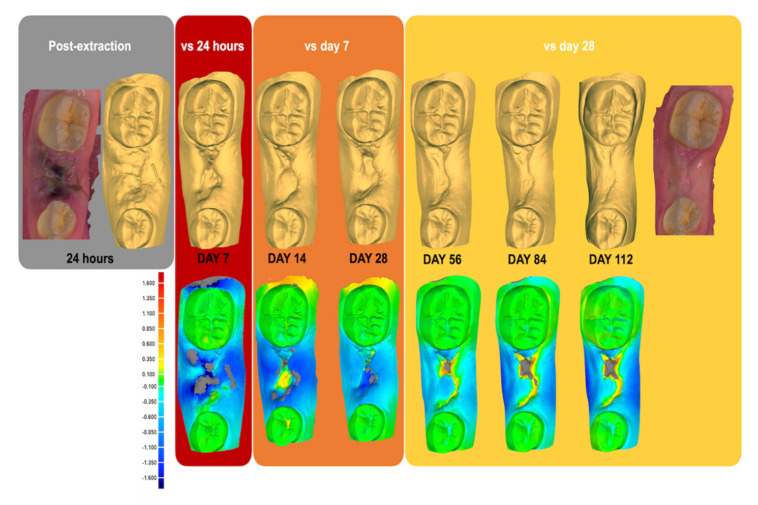
Intraoral scans and 3D analysis demonstrating the progression of residual alveolar ridge remodelling and soft tissue invagination post-extraction of tooth 46 over a period of 112 days, achieved through superimposition and 3D deviation analysis of sequential intraoral scans. The grey colour indicates the absence of data.

**Figure 3:**
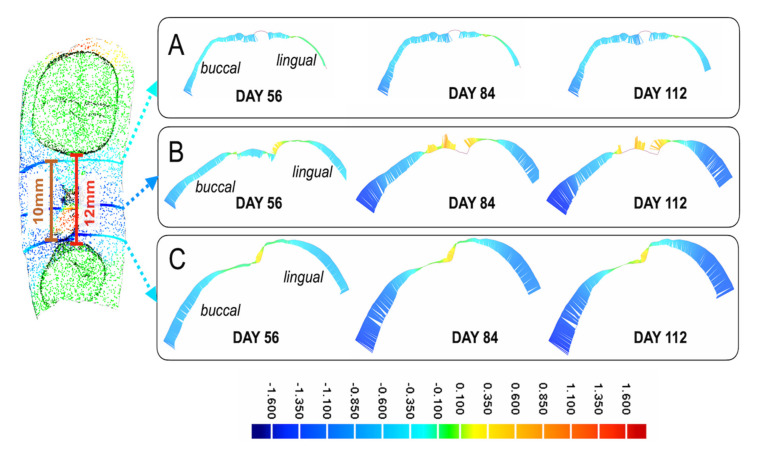
Residual alveolar ridge remodelling assessed through 2D comparison of sequential intraoral scans 112 days post-extraction of mandibular first molar versus the day 28 reference scan. The 2D analysis was performed using sagittal planes across the ridge at three locations: 1 mm from the second molar (**A**); midline of ridge (**B**); and 1 mm from second premolar (**C**).

The first seven days post-extraction demonstrated the greatest dimensional changes to the alveolar ridge (Figure 2) with the mean negative maximum deviation, identified using 3D analysis, being −1.7 mm ± 0.3, when compared to day 0 (Table 1). Over the following twenty-one days and up to day 28, the mean deviation dropped to 1.3 mm ± 0.1, when compared to day 7. Finally, between day 28 and day 112, the mean deviation was 1.3 mm ± 0.2, when compared to day 28, demonstrating plateauing in the progression of ridge remodelling activity over eighty-four days. The 2D analysis (Figure 3 and Table 1) further identified that the largest deviations were present at the buccal region, especially at the midline area of the ridge (B) followed by the approximal area closest to tooth 45 (B), and to a lesser extent the area closest to tooth 47 (C).

## 4. Discussion

The findings of this report identified that the average 3D reduction to the width of the alveolar ridge was −3 mm at four months when compared to one-week data. The highest rate of dimensional changes occurred within the first seven and twenty-eight days post-extraction. The 2D assessment further identified that the greatest dimensional changes occurred in the mid area of the ridge versus the approximal areas of the neighbouring teeth. The findings of this report agree with those of a systematic review that investigated the dimensional changes in the alveolar ridge in the anterior and premolar teeth over a period of three to twelve months [12]. The review identified a 3.87 mm reduction in ridge width, with 1.67 mm and 2.59 mm height losses for the mid-buccal and mid-lingual areas, respectively, compared to 0.64 mm for the approximal areas of the neighbouring teeth. Clinically, the presence of localised pocketing and bleeding on probing at the site of the lower right molar indicated the presence of periodontitis, further modified by a history of smoking. A recent systematic review and meta-analysis has indeed confirmed that high probing pocket depth, smoking, and molar teeth are all associated with a higher risk of tooth loss [13]. Nonetheless, non-surgical periodontal management through scaling and root planning supplemented by the use of adjuncts, such as nutraceutical agents, has been demonstrated to result in a significant reduction in probing depth, bleeding on probing, level of inflammatory mediators, as well as short-term pain in periodontitis patients [14,15]. The presence of extensive subgingival caries involving the furcation area of tooth 46, however, made the tooth unrestorable and complicated the prognosis of any non-surgical periodontal management.

The proposed technique of using intraoral scanning, surface matching, and deviation analyses demonstrated its potential to identify, quantify, and monitor the progression of ridge modelling post-extraction over a period of four months. It offers several advantages and presents some limitations. The advantages of the proposed technique lie in its potential ability to accurately monitor positive and negative changes to soft and hard tissues in a non-invasive manner, with no radiation exposure risk to the patient. Intraoral scanning offers reduced chairside time and improved patient comfort, compared to conventional impression making [7]. Moreover, intraoral scanning in conjunction with cone-beam computed tomography (CBCT), 3D printing, and extraoral face scans can assist in creating a virtual dental patient image and possibly offer a more streamlined and accurate digital workflow for dental care delivery [4,16]. On the other hand, a possible limitation of the technique is the need to ensure that a strict and standardised protocol is followed in acquiring the scans. Inaccurate scans due to inconsistent scanning protocol or missing data can complicate the deviation analyses. Additionally, the accuracy of intraoral scans is dependent on the size of the scan, with dentate full-arch and edentulous arch scans demonstrated to be less accurate than sectional/partial arch scans [17]. Consequently, the assessment of dimensional changes in edentulous arches can be challenging in the absence of reliable reference areas. Finally, data preparation of scans and deviation analyses can be cumbersome and require training and expertise in the use of specialised software and must be accounted for with large-scale application of the technique. Nonetheless, further validation of the proposed technique, as part of future clinical trials with larger sample sizes based on appropriate power calculation and statistical analysis, is needed.

## 5. Conclusions

The 2D and 3D deviation analyses of sequential intraoral scans using a standardised and strict assessment protocol demonstrate the potential of this method as a valuable and supplemental aid to monitor dimensional changes to the residual alveolar ridge over time, overcoming the limitations of more invasive assessment approaches.

## Figures and Tables

**Table 1:** Maximum deviation error calculated over 4 months using 3D and 2D deviation analyses (A,B,C). * Denotes comparison versus day 0 scan. ^#^ Denotes comparison versus day 7. ^+^ Denotes comparison versus day 28. All measurements are in millimetres.

Scan Day	3D Deviation	Molar (A)	Midline (B)	Premolar (C)
Day 2 *	−1.4	−0.7	−0.8	−0.7
Day 3 *	−1.6	−1.4	−1.3	−1.2
Day 7 *	−2	−1.2	−1.9	−1.2
Day 10 ^#^	−1.2	−0.3	−0.9	−0.8
Day 14 ^#^	−1.5	−0.6	−1.4	−1.1
Day 21 ^#^	−1.2	−0.8	−1.0	−1.0
Day 28 ^#^	−1.4	−0.9	−1.3	−1.2
Day 35 ^+^	−1.2	−0.4	−1.1	−0.5
Day 42 ^+^	−1.1	−0.5	−0.9	−0.5
Day 56 ^+^	−1.6	−0.9	−0.9	−0.9
Day 70 ^+^	−1.2	−0.9	−1.2	−1.1
Day 84 ^+^	−1.5	−1.0	−1.4	−1.4
Day 112 ^+^	−1.6	−1.1	−1.5	−1.5
